# Inkjet-Printed Organic Transistors Based on Organic Semiconductor/Insulating Polymer Blends

**DOI:** 10.3390/ma9080650

**Published:** 2016-08-02

**Authors:** Yoon-Jung Kwon, Yeong Don Park, Wi Hyoung Lee

**Affiliations:** 1 Department of Organic and Nano System Engineering, Konkuk University, Seoul 05029, Korea; yjkwon@konkuk.ac.kr; 2 Department of Energy and Chemical Engineering, Incheon National University, Incheon 22012, Korea; ydpark@inu.ac.kr

**Keywords:** polymer blend, organic field-effect transistor, inkjet printing, organic semiconductor, soluble acene, printed electronics

## Abstract

Recent advances in inkjet-printed organic field-effect transistors (OFETs) based on organic semiconductor/insulating polymer blends are reviewed in this article. Organic semiconductor/insulating polymer blends are attractive ink candidates for enhancing the jetting properties, inducing uniform film morphologies, and/or controlling crystallization behaviors of organic semiconductors. Representative studies using soluble acene/insulating polymer blends as an inkjet-printed active layer in OFETs are introduced with special attention paid to the phase separation characteristics of such blended films. In addition, inkjet-printed semiconducting/insulating polymer blends for fabricating high performance printed OFETs are reviewed.

## 1. Introduction

Organic field-effect transistors (OFETs) have received much attention as key components for enabling flexible electronics on plastic substrates [1,2]. Today, the performances of OFETs are comparable to or exceed those of a-Si FETs. However, OFETs have several technological difficulties including bias instability and time dependent degradation of performances [3,4]. Thus, OFETs may not be able to replace traditional high-speed devices that are based on single crystal silicon. In this regard, the merits of OFETs are mainly cost efficiency and plastic compatibility [5]. Considering that cost-effective printing methods are technologically desirable for realizing large-area OFET arrays, all layers (i.e., semiconductor, dielectric, electrode) consisting of OFETs could be fabricated via printing methods [6,7,8,9]. For example, roll-to-roll printing such as gravure or offset printing provides patterned electrodes, semiconductors, and dielectrics [10,11]. Consecutive roll-to-roll printing could therefore reduce the production cost of OFETs. Notwithstanding these benefits of roll-to-roll printing, reducing feature size and registration error are obstacles to the commercialization of roll-to-roll printed OFETs. On the other hand, inkjet printing is technologically advantageous because the pattern can be directly formed by dropping ink onto a desired region [12,13]. In addition, it is an economical method as no waste is generated during the process. Moreover, feature size can be reduced by decreasing the size of the nozzle [14]. Semiconducting layers of OFETs can also be successfully fabricated by inkjet printing with organic semiconductor inks [15], exhibiting excellent device performances comparable to those of OFETs based on spin-cast organic semiconducting layers.

**Figure 1:**
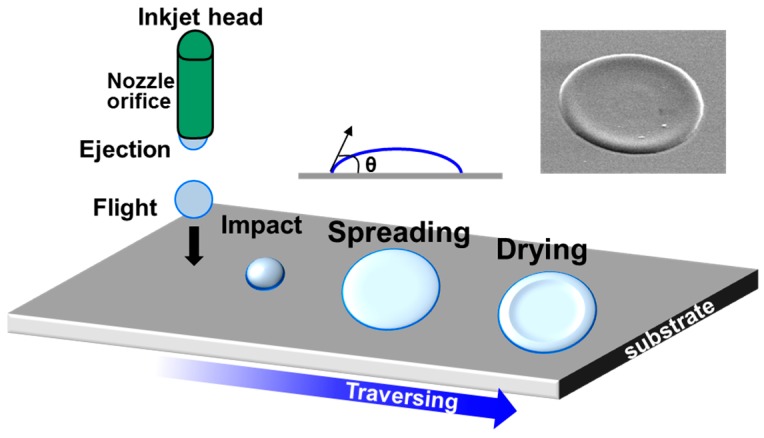
Schematic diagram showing the inkjet printing process. The right/top inset shows scanning electron microscopy (SEM) image of inkjet-printed poly(3,4-ethylenedioxythiophene)/poly(styrenesulfonate) (PEDOT/PSS) electrode [5]. Copyright 2013 American Chemical Society.

The inkjet printing process is schematically shown in Figure 1. Inkjet printable materials should have sufficient ink properties (i.e., surface tension, solvent evaporation rate, and solubility) for successful ejection at the nozzle orifice. After ejection, the ink shoots out of the nozzle and impacts the substrate while it spreads and dries during evaporation of the solvent [5]. Typically, the outward convective flow in the pinned droplet results in a ring stain, as shown in the inset in Figure 1. This coffee ring effect is commonly regarded as an undesirable aspect of inkjet printing, since uniform morphology with reduced ring stain is necessary for inkjet-printed organic semiconductor films. Besides morphology, the crystalline structure of the organic semiconductor is also important for improving the performances of OFETs based on inkjet-printed organic semiconductor film. The type of solvent, concentration of ink, and/or substrate wettability can control the morphology and crystalline structure of inkjet-printed organic semiconductor films. Many efforts have been made to enhance field-effect mobilities of OFETs [15,16,17]. Various review articles have been presented on inkjet-printed OFETs [5,9]. For example, it has been demonstrated that blending an organic semiconductor with an insulating polymer is advantageous for enhancing jetting properties [18]. Since the solubility of organic semiconductors is rather limited and crystallization can occur at the nozzle orifice, adding an insulating polymer can enhance the jetting properties. In addition, such a blend approach is quite appealing for reducing the cost of materials, enhancing mechanical properties, reducing processing steps, and/or increasing device stabilities [19,20,21,22,23,24,25,26,27,28,29,30,31,32,33,34,35,36,37,38,39,40]. To obtain comparable or enhanced device performances, the morphologies and crystalline structures of inkjet-printed organic semiconductor/insulating polymer blend films need to be controlled. Especially, charge transport along the lateral direction should be guaranteed when utilizing organic semiconductor/insulating polymer blend film in the active layer in OFETs [19].

In this review article, inkjet-printed organic transistors based on organic semiconductor/insulating polymer blends are reviewed. The purposes of the blend approach in inkjet printing are as follows: (1) enhancing jetting efficiency; (2) reducing materials cost; (3) increasing uniformity in inkjet-printed deposit; and (4) enhancing device performances. We will introduce topics from solution-processed small molecular semiconductor (namely, soluble acene)/insulating polymer blends to semiconducting/insulating polymer blends. For each work reviewed, phase separation control for guaranteeing charge transport along the lateral direction is also introduced and analyzed.

## 2. Soluble Acene/Insulating Polymer Blends

TIPS-pentacene (6,13-Bis(triisopropylsilylethynyl) pentacene) is a representative soluble acene [41,42,43]. It has high solubility and co-facial stacking with reduced π–π stacking distance. Many studies have utilized TIPS-pentacene as an active layer in OFETs [41]. When TIPS-pentacene/insulating polymer blends are used as an active layer of OFETs, the phase-separated structure typically contains TIPS-pentacene-top, insulating polymer-middle, and TIPS-pentacene-bottom tri-layers [34,44]. Because the conducting channel in this phase-separated structure is guaranteed, many researchers have attempted to utilize TIPS-pentacene/insulating polymer blends as the active layer in OFETs. One of the merits for tri-layer phase-separation is that insulating polymer in the middle can protect the conducting channel (TIPS-pentacene-bottom layer) from atmospheric air. Thus, environmental stabilities of OFETs are greatly enhanced. When designing the inkjet printing of TIPS-pentacene/insulating polymer, the printability and microstructure of the ring-like deposits should be considered.

**Figure 2:**
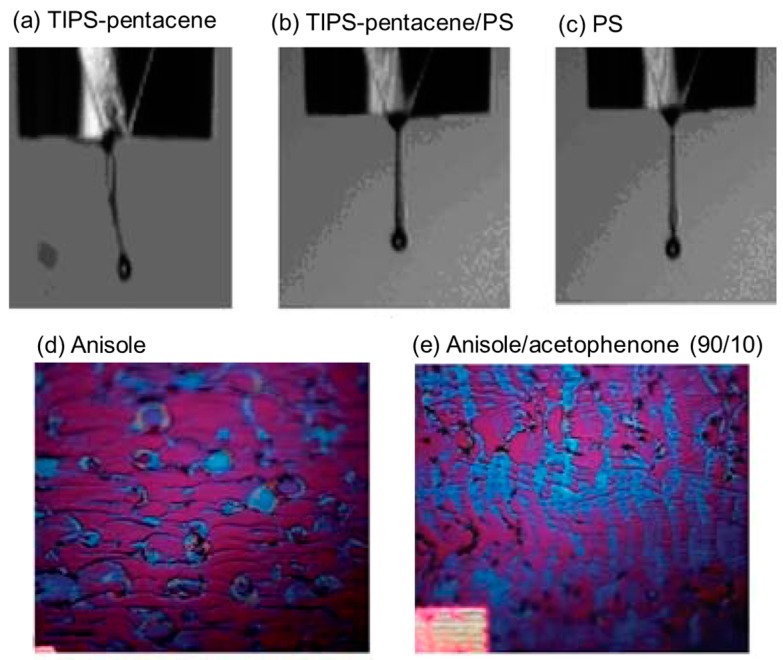
Droplet profiles of (**a**) 6,13-Bis(triisopropylsilylethynyl) pentacene (TIPS-pentacene, 1 g/dL in anisole); (**b**) TIPS-pentacene/polystyrene (PS) blends (1:1 *w*/*w*, 1 g/dL in anisole); and (**c**) PS (0.5 g/dL in anisole. Optical microscopy images of inkjet-printed TIPS-pentacene/PS blend films using (**d**) anisole solution or (**e**) anisole/acetophenone (90/10) mixed solution [18]. Copyright 2010 Royal Society of Chemistry.

Madec et al. added amorphous polystyrene (PS) to TIPS-pentacene ink to enhance the jetting properties of the ink [18]. Such a blend approach is beneficial for the formation of uniform films. Droplet profiles of TIPS-pentacene (1 g/dL in anisole), TIPS-pentacene/PS blends (1:1 *w*/*w*, 1 g/dL in anisole), and PS (0.5 g/dL in anisole) are shown in Figure 2a–c. For TIPS-pentacene ink, a distorted ligament with satellites is detected. This is typical in low-viscosity Newtonian fluids. When PS is added to TIPS-pentacene ink, the stabilization of ligament and coalescing of satellites can lead to greater control of the droplet generation process (Figure 2b). This clearly indicates that the added insulating polymer is beneficial for the inkjet drop ejection process. When TIPS-pentacene/PS blend solution in anisole is inkjet-printed, the coffee staining effect induces the formation of artifacts at the edge of the printed deposits (Figure 2d). However, in the case of anisole/acetophenone (90/10), a uniform deposit with high crystallinity is formed due to the reduced convective flow. Because acetophenone has a high boiling point while TIPS-pentacene has a limited solubility in this solvent, the coffee staining effect is decreased but crystallinity is increased significantly (Figure 2e).

**Figure 3:**
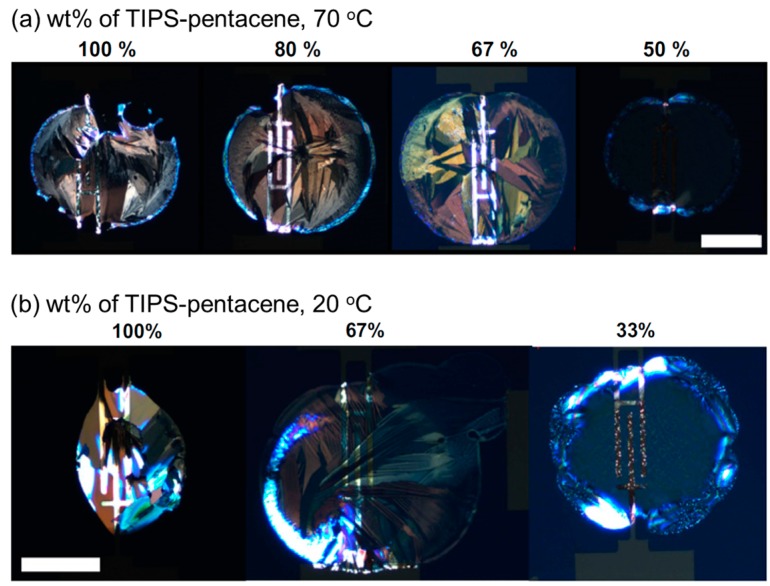
Polarized optical microscopy images showing transition of TIPS-pentacene/PS blend films with increasing wt % of TIPS-pentacene: (**a**) Substrate temperature at 70 °C; (**b**) Substrate temperature at 20 °C. Scale bar: 100 μm [45]. Copyright 2011 Elsevier.

The TIPS-pentacene/insulating polymer blend was used for examining the crystallization behavior of TIPS-pentacene in insulating polymer. Vertical phase-separation in spin-cast blend film has been demonstrated by several research groups [19,34,44]. The morphologies and structures of inkjet-printed TIPS-pentacene/PS blend films with various blend ratios have been examined by Li et al. [45]. Figure 3a,b show polarized optical microscopy images of the transition of TIPS-pentacene/PS blend films with increasing wt % of TIPS-pentacene at substrate temperatures of 70 °C and 20 °C, respectively. When pure TIPS-pentacene in tetralin was used for inkjet-printing, the depinning of the droplets resulted in TIPS-pentacene deposit with an irregular shape (Figure 3a, 100%). An increased content of PS and well-resolved TIPS-pentacene crystals with uniform morphologies were observed (80%, 67%). However, further increasing the content of PS to 50% resulted in small crystals at the edge of the ring-like deposit. When the substrate temperature was decreased to 25 °C, the drying time was increased and the contact line depinning was magnified (Figure 3b, 100%). Because tetralin—with a high boiling point—was used as the solvent, substrate heating was necessary to increase the solvent evaporation rate and decrease the contact line depinning.

**Figure 4:**
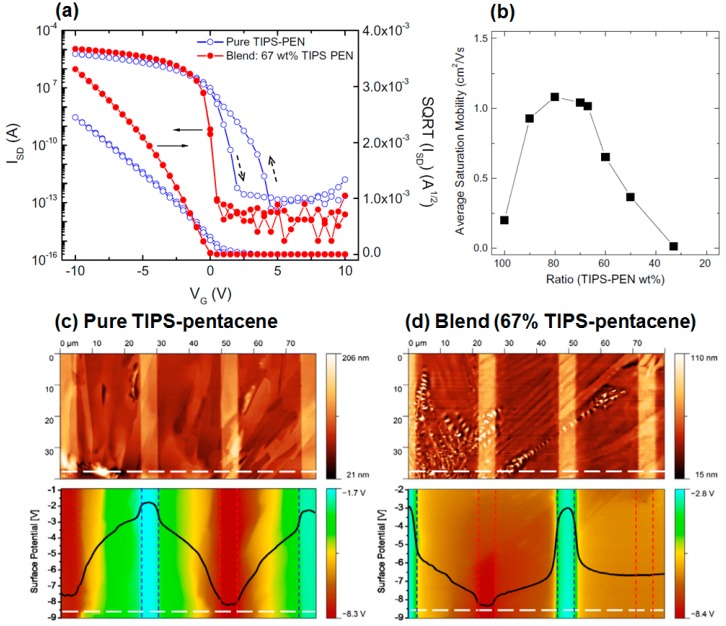
(**a**) Transfer characteristics in saturation regime (*V*_DS_ = −10 V) for field-effect transistors (FETs) based on pure TIPS-pentacene (open circles in blue) and TIPS-pentacene/PS blend of 67 wt % TIPS-pentacene (solid circles in red); (**b**) Average field-effect mobilities as a function of TIPS-pentacene weight ratios in FETs with TIPS-pentacene/PS blend film. 2D topography and corresponding surface potential images were measured with scanning Kelvin probe microscopy (SKPM) of (**c**) pure TIPS-pentacene film or (**d**) TIPS-pentacene/PS blend film (67 wt % TIPS-pentacene) [45]. Copyright 2011 Elsevier.

OFETs with blend film (67% of TIPS-pentacene) exhibited superior electrical properties to those of FETs with pure TIPS-pentacene film (Figure 4a). Field-effect mobility showed a maximum value at the TIPS-pentacene ratio of 67%–80% (Figure 4b). Interestingly, a low subthreshold slope (sharp turn-on) in the blend OFETs is noticeable. This can be explained by the contact resistance which might have originated from the phase-separated insulating polymer (here, PS). To investigate the validity of this assumption, scanning Kelvin probe microscopy (SKPM) was used to detect any potential drop at the contact region. Figure 4c,d show comparative topography and surface potential images. The bright regions in the topography images correspond to the Au source/drain electrodes. The blend sample exhibited a higher potential drop at the contact region compared to that of the pure TIPS-pentacene sample. This implied the existence of hole trapping at the contact region of the blend OFETs, thereby contributing to a sharp turn-on as shown in Figure 4a.

**Figure 5:**
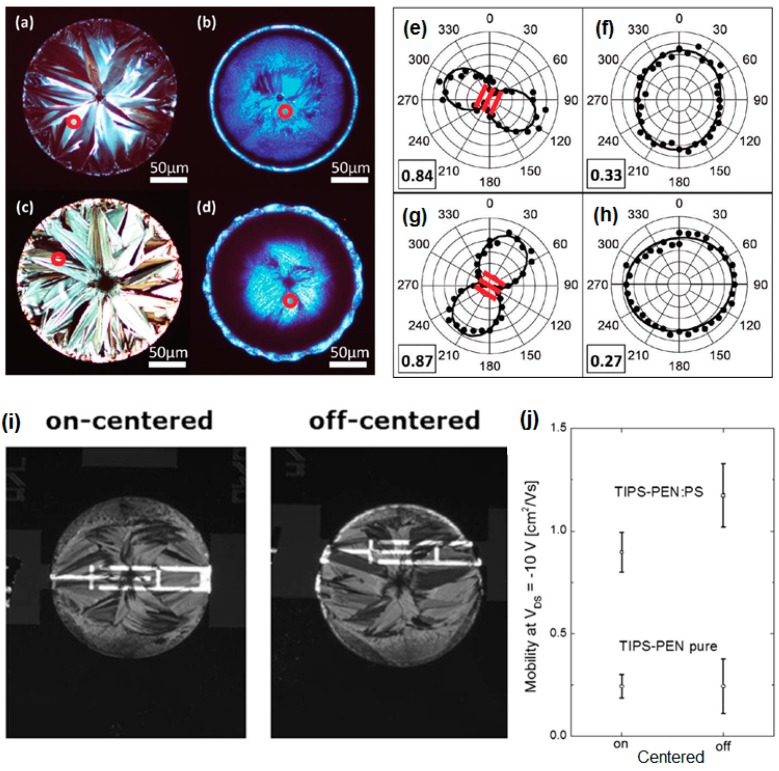
Polarized optical microscopy images of inkjet-printed droplets containing (**a**); (**b**) TIPS-pentacene and (**c**); (**d**) TIPS-pentacene/PS blend films deposited on (**a**); (**c**) pentafluorobenzenethiol (PFBT)-treated Au and (**b**); (**d**) trichlorophenylsilane (TCPS)-treated silicon substrates; (**e**–**h**) Raman intensity of the C–C ring stretch mode as a function of polarization angle taken from red circle points in (**a**–**d**) [46]. Copyright 2011 American Chemical Society; (**i**) Polarized optical microscopy images of ink-jet printed TIPS-pentacene/PS blends: on-centered (left) and off-centered (right); (**j**) Average field-effect mobility of FETs based on on- and off-centered ink-jet printed deposits [47]. Copyright 2013 Elsevier.

The crystallization characteristics of the TIPS-pentacene/PS blend could also be affected by the surface characteristics of the Au source/drain electrodes [46]. Polarized optical microscopy images of the inkjet-printed deposits of the TIPS-pentacene and TIPS-pentacene/PS blend films on pentafluorobenzenethiol (PFBT) treated Au electrodes are shown in Figure 5a,c, respectively. Polarized optical microscopy images of the inkjet-printed deposits of the TIPS-pentacene and TIPS-pentacene/PS blend films on trichlorophenylsilane (TCPS) treated SiO_2_/Si substrates are shown in Figure 5b,d, respectively. Highly crystalline TIPS-pentacene crystals were predominantly observed in the PFBT treated Au electrodes. The Raman intensity of the C–C ring stretch mode as a function of the polarization angle as shown in Figure 5e–h taken from the red circled points in Figure 5a–d supports the presence of anisotropic TIPS-pentacene crystals on the PFBT treated Au and isotropic TIPS-pentacene crystals on the TCPS treated SiO_2_/Si. Importantly, the added PS did not disturb the growth of the highly oriented TIPS-pentacene crystals. Instead, the TIPS-pentacene/PS blend led to a uniform film with reduced variations in molecular orientation. Thus, the field-effect mobility of OFETs based on the TIPS-pentacene/PS blend was increased to more than 0.72 cm^2^/Vs, whereas the OFETs based on single TIPS-pentacene exhibited mobility of 0.22 cm^2^/Vs. Kjellander et al. enhanced the electrical properties of TIPS-pentacene/PS OFETs by using off-centered inkjet printing [47]. Because the crystallization of TIPS-pentacene in the blend started at the edge of the ring-like droplet, the TIPS-pentacene crystals met at the center (Figure 5i). Thus, the OFETs based on off-centered printing led to higher field-effect mobility (1.1 cm^2^/Vs) than that of the OFETs based on on-centered printing (Figure 5j). Unipolar circuitry building blocks and a radio-frequency identification (RFID) transponder can also be fabricated using off-centered inkjet-printed TIPS-pentacene/PS blends. 

**Figure 6:**
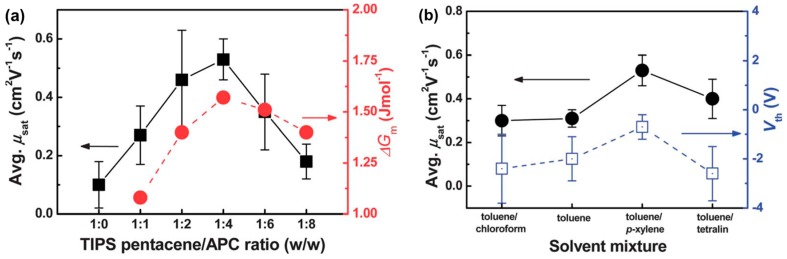
(**a**) Average field-effect mobility (left) of FETs based on inkjet-printed TIPS-pentacene/amorphous polycarbonate (APC) blends (left) and Gibbs free energy of mixing (right) as a function of blending ratio; (**b**) Average mobility and threshold voltage of FETs based on inkjet-printed TIPS-pentacene/APC blends with respect to solvent mixture [48]. Copyright 2013 Royal Society of Chemistry.

Cho et al. used amorphous polycarbonate (APC) to blend with TIPS-pentacene to fabricate FETs [48]. They calculated the Flory–Huggins parameters of several insulating polymers (PS, APC, and poly(α-methlystyrene) (PαMS)) and TIPS-pentacene, and found that APC has the highest value for phase-separation. Thus, they used TIPS-pentacene/APC blends for fabricating FETs and changed the blending ratio while fixing the total concentration. The blend ratio of 1:4 (TIPS-pentacene/APC) was found to be the optimum condition to exhibit high field-effect mobility (Figure 6a). Interestingly, this condition exactly matched the maximum Gibbs free energy of mixing, indicating that thermodynamically driven strong segregation is a reason for the observed results. 

**Figure 7:**
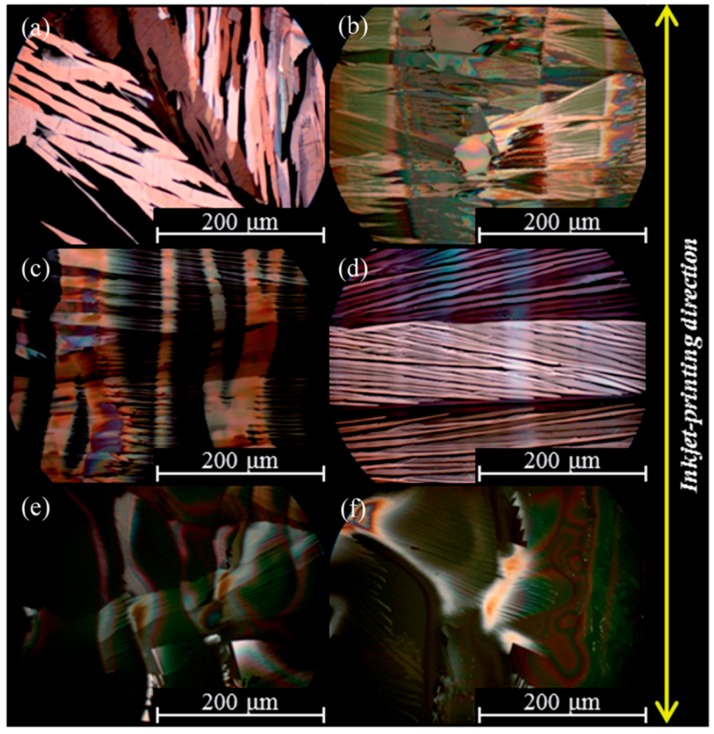
Polarized optical microscopy images of (**a**) inkjet-printed TIPS-pentacene and (**b**–**f**) TIPS-pentacene/APC blends (1:1), (1:2), (1:4), (1:6), (1:8), respectively [48]. Copyright 2013 Royal Society of Chemistry.

**Figure 8:**
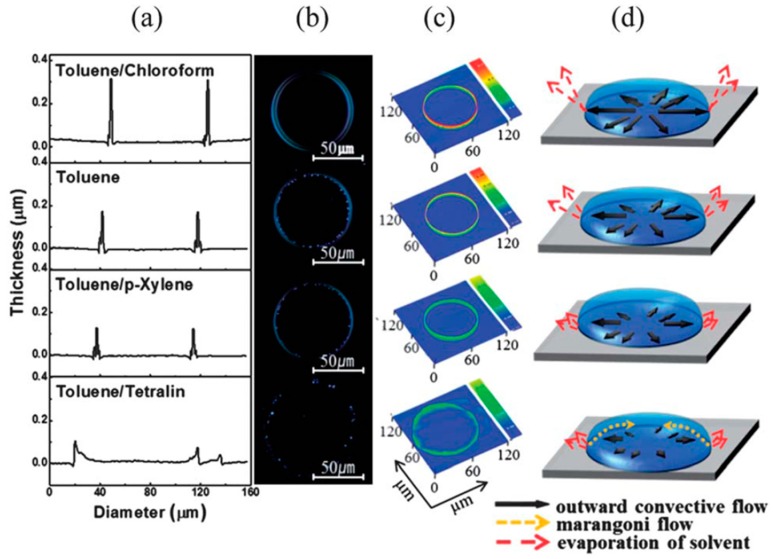
Mixed solvent approach in TIPS-pentacene/APC blends: (**a**) Profile images; (**b**) polarized optical microscopy images; (**c**) 3D profile images; and (**d**) schematic drawings of inkjet-printed TIPS-pentacene/APC droplets [48]. Copyright 2013 Royal Society of Chemistry.

Polarized optical microscopy images of the inkjet-printed lines of TIPS-pentacene and TIPS-pentacene/APC blends are shown in Figure 7a–f, respectively. For this experiment, it was ensured that the inkjet-printed lines were perpendicular to the source/drain electrodes by considering the crystallization characteristics of TIPS-pentacene. Pure TIPS-pentacene in the inkjet-printed lines yielded inclined crystals (Figure 7a). Importantly, well-oriented needle-shaped crystals in parallel directions occurred at the blend ratio of 1:4 (Figure 7d). The further increase of APC content resulted in the formation of plate-like wavy crystals (Figure 7e,f). These morphological evolutions were related to the calculation of the Gibbs free energy of mixing that showed the highest value in the blend ratio of 1:4 (Figure 6a). Morphological and theoretical studies supported the obtained high mobility at the blend ratio of 1:4. The mixed solvent approach in the inkjet-printed TIPS-pentacene/APC blends is shown in Figure 8. The coffee staining effect appeared in all solvent compositions. Especially, when a solvent with a low boiling point (e.g., chloroform) was added to toluene, the enhanced convective flow resulted in large edge walls. On the other hand, when a solvent with a high boiling point (e.g., *p*-xylene) was added, the coffee staining effect decreased. Interestingly, when tetralin was added, the solvent evaporation at the edge was very slow, leading to an irregular edge profile confirmed by the surface profile and polarized optical microscopy image. Accordingly, the use of the toluene/*p*-xylene mixed solvent resulted in the highest field-effect mobility in the application of the active layer in OFETs (Figure 6b). This result indicated that a mixed solvent approach to control the inkjet-printed single TIPS-pentacene droplets was also effective in the blend system consisting of TIPS-pentacene and insulating polymer.

**Figure 9:**
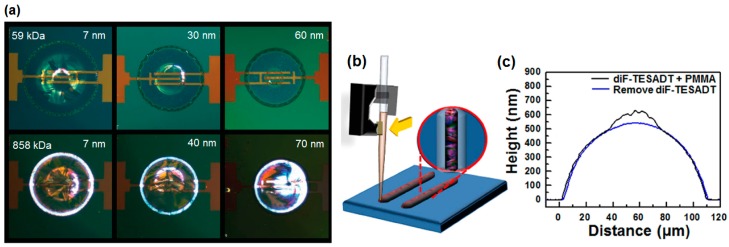
(**a**) Polarized optical microscopy images of TIPS-pentacene deposits on poly(α-methlystyrene) (PαMS) layers of various thicknesses. Top: 59 kDa (1, 30, 60 nm), Bottom: 858 kDa (7, 40, 70 nm). Substrate temperature was kept at 70 °C [49]. Copyright 2010 Wiley; (**b**) Schematic of printing process of 2,8-difluoro-5,11-bis(triethylsilylethynyl)anthradithiophene (diF-TESADT)/poly(methyl methacrylate) (PMMA) blend using picoliter fluidic dispensing system; (**c**) Surface profiles of diF-TESADT/PMMA blend before and after selective removal of diF-TESADT [51]. Copyright 2015 Nature Publishing Group.

Although the inkjet-printing of TIPS-pentacene/insulating polymer blend is a rational approach to enhance the electrical properties of OFETs, inkjet printing TIPS-pentacene solution on insulating polymer can yield similar results [49]. Polarized optical microscopy images of TIPS-pentacene deposits on PαMS layers with various thicknesses are shown in Figure 9a. Two types of PαMS with different molecular weights (59 and 858 kDa) were used. Because the solvent tetralin for inkjet printing of TIPS-pentacene dissolved PαMS, the mixing of TIPS-pentacene and PαMS occurred in the TIPS-pentacene droplet. A self-aligned bank was formed due to the coffee staining effect (Figure 9a). The thickness and molecular weight of PαMS affected the final morphologies of the dried TIPS-pentacene/PαMS deposits. Especially, a highly crystalline characteristic of the TIPS-pentacene crystals was observed when 30 nm-thick PαMS (59 kDa) and 7 nm-thick PαMS (858 kDa) were used. This in turn resulted in the high field-effect mobility (0.7–0.8 cm^2^/Vs) of the corresponding OFETs. The method used in this approach could enhance the alignment of TIPS-pentacene and uniformity in the performances of the OFETs, mainly due to the formation of the self-aligned bank.

When 2,8-difluoro-5,11-bis(triethylsilylethynyl)anthradithiophene (diF-TESADT) was used for soluble acene [50], the blend of the diF-TESADT/insulating polymer typically led to a bilayer structure of diF-TESADT-top/insulating polymer-bottom [24]. This is because diF-TESADT exhibits low surface energy with a tendency to segregate at the air-film surface. Lee et al. used a diF-TESADT/poly(methyl methacrylate) (PMMA) blend and a picoliter fluidic dispenser similar to an inkjet printer to fabricate a diF-TESADT-top/PMMA-bottom bilayer structure [51]. Schematic drawings of the printing process and corresponding printed lines are shown in Figure 9b. Surface profiles of the diF-TESADT/PMMA blend before and after the selective removal of diF-TESADT are shown in Figure 9c, demonstrating that a diF-TESADT-top/PMMA-bottom bilayer structure is spontaneously formed during printing. They utilized phase-separated diF-TESADT as a semiconductor and phase-separated PMMA as a dielectric and succeeded in fabricating both the semiconductor and dielectric layers via a one-step printing process [22].

## 3. Semiconducting/Insulating Polymer Blends

The phase-separation characteristics of the films of semiconducting/insulating polymer blends also show potential for use in the active layer of OFETs [25]. However, the phase-separations of polymer/polymer blends are complicated phenomena [22,52] and can be affected by thermodynamic and kinetic parameters. Thermodynamically, three component phase diagrams consisting of a semiconducting polymer, an insulating polymer, and a solvent should be considered when examining the phase-separation characteristics. Although it is beneficial for the application of polymer blends in OFETs, vertical phase-separation can occur under well-controlled conditions with the kinetic control of solvent evaporation as an indispensable step in obtaining a vertically phase-separated structure [19,32]. For example, the laterally/vertically phase-separated structures of polymer blends are affected by the choice of solvent because of the effect of the evaporation speed of solvent [53]. In addition, substrate wettability can influence the phase-separation morphology and the structure of polymer blends [36]. Qiu et al. successfully fabricated a poly(3-hexylthiophene) (P3HT)-top/PMMA-bottom bilayer structure by spin-casting a P3HT/PMMA blend solution onto a hydrophilic silicon substrate [4]. Thermodynamically, the reason for this behavior is that P3HT exhibits a lower surface energy than PMMA. Although the same P3HT/PMMA blend was used, the PMMA-top/P3HT-bottom bilayer structure is instead fabricated onto the hydrophobic substrate [7]. Several strategies have been suggested to induce vertical phase separation in semiconducting/insulating polymer blends. Heriot et al. proposed that lateral phase separation is much more common in polymer/polymer blends due to their Marangoni-like instability [52].

**Figure 10:**
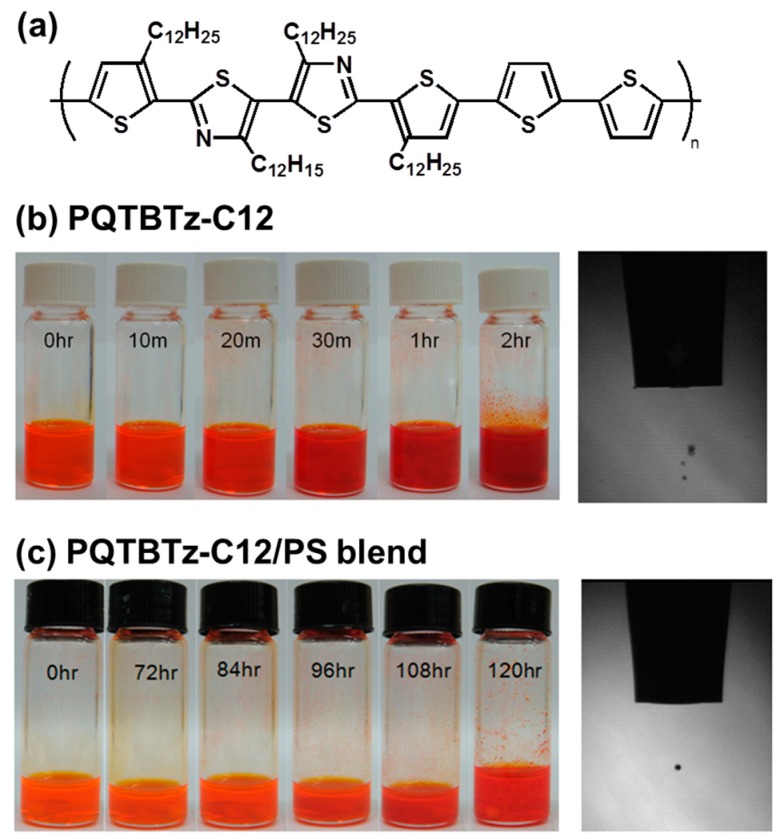
(**a**) Chemical structure of poly(didodecylquaterthiophene-alt-didodecylbithiazole) (PQTBTz-C12); Photographic images of solution (left), droplet profile (right) of (**b**) PQTBTz-C12 and droplet profile of (**c**) PQTBTz-C12/PS blend. The photographic images on the left were collected at various stages of aging [54]. Copyright 2016 Wiley.

In inkjet printing, the vertical phase-separation of polymer/polymer blends is difficult to achieve due to the dynamic change of flow in the printed droplets. However, the use of semiconducting/insulating polymer blends for fabricating an active layer in OFETs has unique merits. Kwak et al. used poly(didodecylquaterthiophene-alt-didodecylbithiazole) (PQTBTz-C12)/PS blends for the formation of an active layer by inkjet printing [54]. The chemical structure of PQTBTz-C12 is shown in Figure 10a [55]. When a single PQTBTz-C12 solution is inkjet-printed, the ejected droplets at the nozzle contain distorted ligaments and several satellites (Figure 10b, right). This is because PQTBTz-C12 has low solubility in solvent (here, chlorobenzene). A change of color occurs in the PQTBTz-C12 solution at various stages of aging (Figure 10b, left). As aging was increased, the aggregation of PQTBTz-C12 occurred in 20–30 min. This reduced the solubility and crystallization behavior of PQTBTz-C12, typically inducing nozzle clogging during inkjet printing. When PS was added to the PQTBTz-C12 solution, no satellite was detected (Figure 10c, right). The aging experiment with the PQTBTz-C12/PS blend demonstrated the long-term stability of the blend solution (Figure 10c, left).

**Figure 11:**
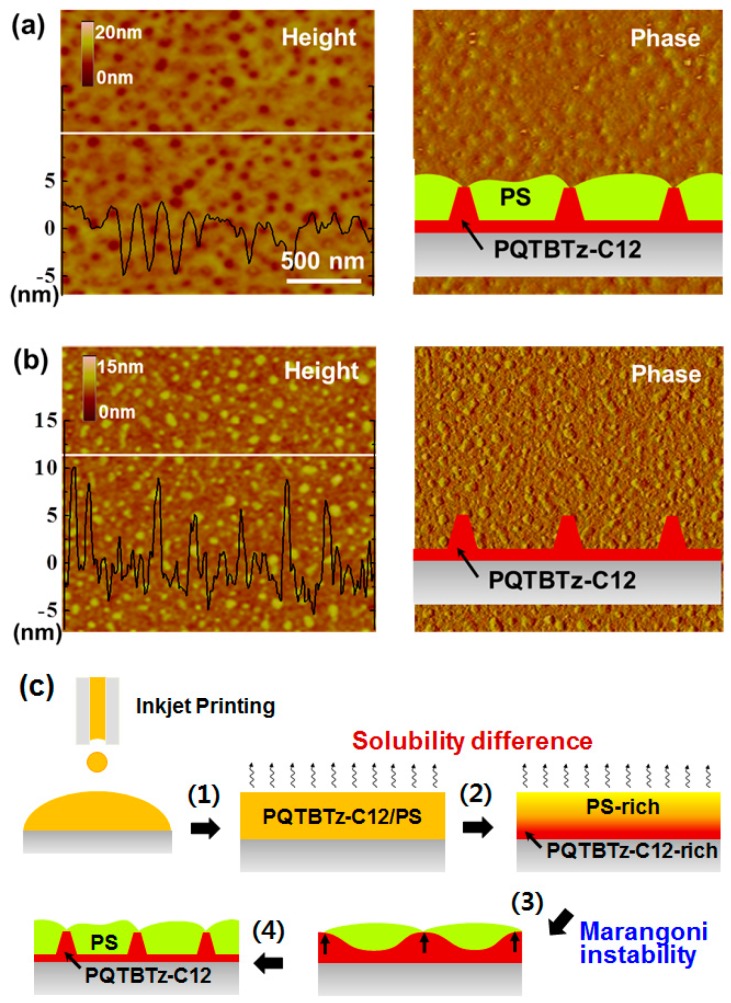
Atomic force microscopy (AFM) height (left) and phase (right) images of (**a**) inkjet-printed PQTBTz-C12/PS blend film and (**b**) remaining PQTBTz-C12 film after selective removal of PS. Insets show schematic drawings; (**c**) Schematic representations showing formation of PQTBTz-C12/PS blend film during drying of inkjet-printed droplets [54]. Copyright 2016 Wiley.

The inkjet-printed morphology of the PQTBTz-C12/PS blend is shown in Figure 11a. The regular isolated holes in the image of the height of the blend indicate that the phase-separation characteristics are complicated. By etching the PS-rich region with cyclohexane, it is possible to examine the underlying PQTBTz-C12 layer (Figure 11b). A continuous PQTBTz-C12 layer with island-like domains was detected. The schematic representation (inset of Figure 11a) shows the phase-separated structure obtained in this study. Although a PS-top/PQTBTz-C12-bottom bilayer structure was formed, the vertical phase-separation was not perfect. The cause of these vertical and lateral phase-separations is illustrated in Figure 11c, which shows that when the PQTBTz-C12/PS blend was inkjet-printed, PQTBTz-C12 with low solubility was solidified first and PS-top/PQTBTz-C12-bottom was formed in the transient wetting layer. However, the Marangoni flow led to the extrusion of the PQTBTz-C12 bottom layer into the PS region, thereby leading to the formation of a dual phase-separated structure with a laterally phase-separated PS layer at the top and a vertically phase-separated PQTBTz-C12 layer at the bottom. When the electrical properties of the OFETs based on inkjet-printed PQTBTz-C12/PS were measured, a field-effect mobility comparable to that of single PQTBTz-C12 was obtained. This was due to the continuous PQTBTz-C12 layer that was phase-separated first using the inkjet printing blend solution. It is important to note that the environmental and electrical stabilities of the PQTBTz-C12/PS OFETs were superior to those of the single PQTBTz-C12 OFETs. The PS layer phase-separated at the top might have protected the PQTBTz-C12 bottom layer.

**Figure 12:**
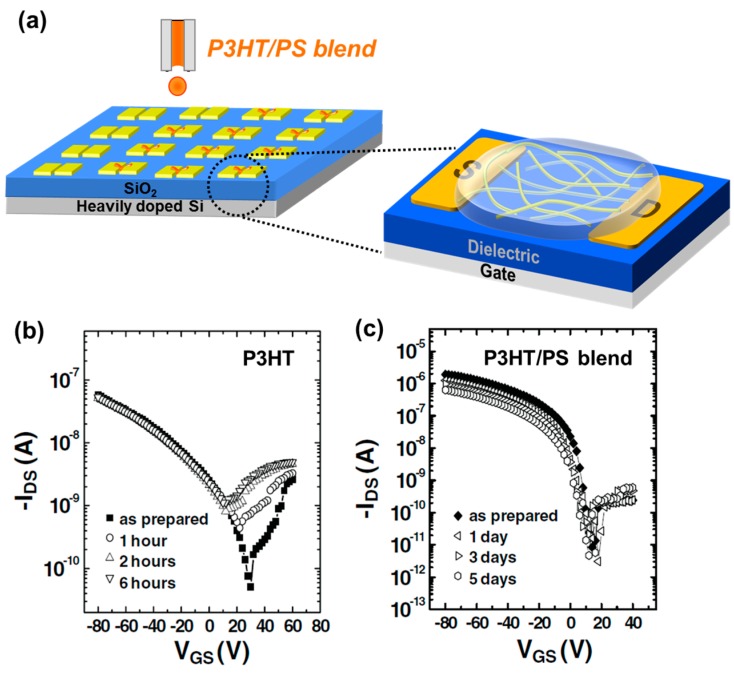
(**a**) Schematic showing inkjet printing of poly(3-hexylthiophene) (P3HT)/PS blend and formation P3HT nanofibers embedded in PS; Environmental stabilities of FETs based on P3HT (**b**) and P3HT/PS (20:80) blend (**c**). Mixed solvent with chlorobenzene (CB) and cyclohexanone (CHN) (80:20) was used to prepare P3HT/PS blend solution [56]. Copyright 2010 Wiley.

Other phase-separated structures for guaranteeing lateral charge transport are necessary in semiconducting/insulating polymer blends due to the limited availability of vertical phase-separation in polymer/polymer blends. Qiu et al. proposed that the change of solubility in the P3HT/PS blend could produce a phase-separated structure with P3HT nanowires embedded in the insulating polymer [28,29,30]. They achieved extremely low percolation behavior by utilizing the P3HT nanowires and a phase-separated PS protected active channel. Furthermore, the environmental stability was enhanced. Lim et al. adopted this technique to produce a phase-separated structure with P3HT nanowires embedded in PS by inkjet printing P3HT/PS blends (Figure 12a) [56]. They found that the addition of non-solvent (cyclohexanone, CHN) in the main solvent (chlorobenzene, CB) yielded the formation of P3HT nanowires in a PS matrix and that the use of a P3HT/PS blend was beneficial for increasing the environmental stability of OFETs (Figure 12b,c, respectively).

**Figure 13:**
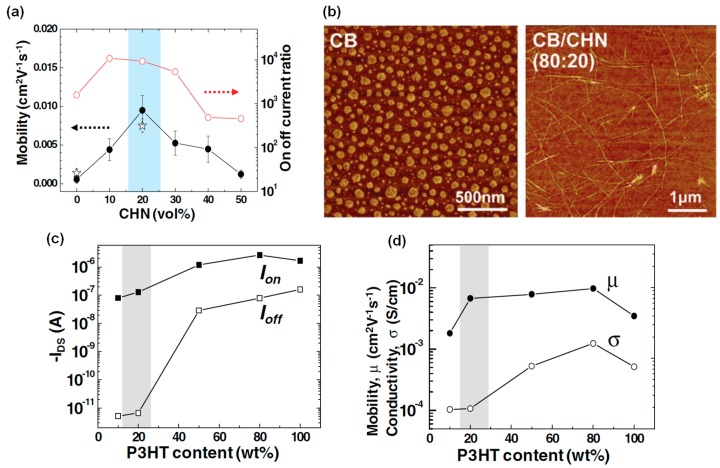
(**a**) Field-effect mobility and *I*_on_/*I*_off_ of P3HT/PS (20:80) FETs as a function of CHN composition in CB/CHN mixed solvent; (**b**) AFM phase images of inkjet-printed P3HT/PS blend films from CB (left) and CB/CHN (80:20) mixed solvent (right); (**c**) *I*_on_/*I*_off_ and (**d**) field-effect mobility/conductivity as a function of P3HT content [56]. Copyright 2010 Wiley.

They examined the role of CHN in P3HT/PS (20:80) FETs. The field-effect mobility and *I*_on_/*I*_off_ of P3HT/PS (20:80) FETs as a function of the CHN composition in CB/CHN mixed solvent are shown in Figure 13a. Clear increases in electrical properties with the addition of 20% CHN were detected. The reason for this behavior was revealed by examining the morphologies of the P3HT/PS blend films. CB led to isolated P3HT aggregates while the CB/CHN mixed solvent resulted in P3HT nanowires becoming embedded in the PS matrix (Figure 13b). The P3HT nanowires formed in PS were extremely beneficial for lateral charge transport. A high on:off current ratio (*I*_on_/*I*_off_) in P3HT/PS FETs compared to P3HT FETs is of interest for further study. The *I*_on_/*I*_off_ and field-effect mobility/conductivity as a function of the P3HT content are shown in Figure 13c,d, respectively. Although the on-current was increased slightly, the off-current was increased abruptly with increasing P3HT content. The reason for the significant increase in the off-current was due to the increased bulk conductivity. Thus, the increase of *I*_on_/*I*_off_ observed in the P3HT/PS (20:80) FETs was driven by a decrease in bulk conductivity. In their study, Lim et al. successfully demonstrated that inkjet-printed semiconducting/insulating polymer blends are also beneficial for increasing *I*_on_/*I*_off_ of FETs [56].

## 4. Conclusions

We reviewed recent advances in inkjet-printed organic transistors based on organic semiconductor/insulating polymer blends. The inkjet printing process was described to provide understanding of the drying behavior of inkjet-printed polymer blends. Soluble acene/insulating polymer blends and semiconducting/insulating polymer blends are attractive inks for enhancing jetting properties, film uniformity, and device performances of OFETs compared to single organic semiconductor inks. The enhanced electrical properties can be understood by considering the morphologies and structures of the blend films under the drying condition of inkjet-printed droplets. The use of mixed solvent is a rational approach to control the phase-separation characteristics and/or enhance the crystallinity of organic semiconductors. In OFETs based on inkjet-printed organic semiconductor/insulating polymer blends, the appropriate blending of insulating polymer can influence the crystallization characteristics of an organic semiconductor, while the insulating polymer plays a role as a dielectric layer or protective layer from atmospheric water and oxygen. Here, the control of phase separation to ensure lateral charge transport is a key to obtaining the suitable functions of devices while increasing their electrical properties.
